# Loving-kindness meditation (LKM) modulates brain-heart connection: An EEG case study

**DOI:** 10.3389/fnhum.2022.891377

**Published:** 2022-09-01

**Authors:** GoonFui Wong, Rui Sun, Jordana Adler, Kwok Wah Yeung, Song Yu, Junling Gao

**Affiliations:** ^1^Neuroscience for Education Laboratory, Faculty of Education, The University of Hong Kong, Pokfulam, Hong Kong SAR, China; ^2^Department of Rehabilitation Sciences, Hong Kong Polytechnic University, Hung Hom, Hong Kong SAR, China; ^3^Interdisciplinary Research Institute at Shasta (IRIS), Eugene, OR, United States; ^4^The Buddha Dharma Centre of Hong Kong Limited, Hong Kong, Hong Kong SAR, China; ^5^Shenzhen EEGSmart Technology Co., Ltd., Shenzhen, China; ^6^Buddhism and Science Research Laboratory, Centre of Buddhist Studies, The University of Hong Kong, Pokfulam, Hong Kong SAR, China

**Keywords:** electroencephalogram (EEG), loving-kindness meditation (LKM), single-channel EEG, theta power, brain-heart connectivity, LKM self-report

## Abstract

Loving-Kindness Meditation (LKM) is an efficient mental practice with a long history that has recently attracted interest in the fields of neuroscience, medicine and education. However, the neural characters and underlying mechanisms have not yet been fully illustrated, which has hindered its practical usefulness. This study aimed to investigate LKM from varied aspects and interactions between the brain, the heart, and psychological measurements. A Buddhist monk practitioner was recruited to complete one 10-min LKM practice, in between two 10-min resting tasks (pre- and post-resting) per experimental run. Two sets of single-channel wearable EEG devices were used to collect EEG data (placed at Fz and Pz) and heart rate simultaneously. A self-report evaluation was conducted to repeatedly record the comprehensive performance of mind and body in each session. EEG data were preprossessed and analyzed by EEGlab. Further statistics were made by SPSS. Spectrum analysis showed a significant increase of theta power (Fz: *t* = −3.356; *p* = 0.002; Pz: *t* = −5.199; *p* < 0.001) and decrease of heart rate between pre- and post-resting tasks (*t* = 4.092, *p* < 0.001). The analysis showed a negative correlation between theta power and heart rate (Fz: *r* = −0.681, *p* < 0.001; Pz: r = −0.384, *p* = 0.008), and a positive correlation between theta power and the self-designed report score (Fz: *r* = 0.601, *p* < 0.001). These findings suggest that LKM is accompanied by significant neurophysiological changes, mainly an increase in slower frequencies, such as theta, and a decrease in heart rate. More importantly, subjective psychological assessments were also correlated with objective neurophysiological measurements in a long-term meditator participant. During LKM meditation, this connection was stronger. The results of this case report have promising implications for LKM practice in daily life.

## Introduction

Psychological wellbeing plays a critically important role in contemporary society, and researchers keep exploring more feasible therapeutic ways to improve mental health (Al-Ghabban, 2018; Allen et al., 2021; Don et al., 2022). After decades of clinical and neuroscientific studies, meditation and mindfulness became popular and gained traction in both scientific and general communities (Boellinghaus et al., 2013, 2014; Le Nguyen et al., 2019; Agrawal and Sahota, 2022; Don et al., 2022). Solid evidence has shown that mindfulness can relieve stress and improve psychological health. Mindfulness is one of the various meditation traditions. Since different meditation techniques may focus on training different mental characters (Lumma et al., 2015; Colzato and Kibele, 2017; Bhanushali et al., 2020; Roca et al., 2021). Different methods of EEG data analysis may result in further conclusions on meditation effectiveness. Moreover, there could be a non-linear trajectory between brain activity and behavioral assessment (Shaw and Routray, 2016, 2018; Gupta et al., 2018; Britton, 2019). Future research is needed to characterize the nature of the many types of meditation (Dahl et al., 2015; Lindahl et al., 2017).

Loving-Kindness Meditation (LKM) is one of the most established Buddhist practices, aiming to generate positive emotions toward oneself and others (Fredrickson et al., 2008; Hutcherson et al., 2008; Cohn and Fredrickson, 2010). LKM training enables individuals to better control their minds and enhance their focus and concentration (Kabat-Zinn and Hanh, 2009). When practicing LKM, individuals need to train the mind to flow smoothly and naturally while enhancing the quality of awareness (Anālayo, 2019). LKM is a cultivation of the sentiments of love, benevolence, kindness, affection, friendship, and goodwill (Fredrickson et al., 2008). LKM emphasizes empathy more than vipassana, i.e., mindfulness, which emphasizes focused attention on specific objects such as breathing. Previous studies may have this difference (Liu et al., 2020).

Several studies and review papers have demonstrated the effectiveness of LKM on symptom improvement for psychiatry disorders, including relieving pain, increasing social connection, reducing anger, hostility, depression, and anxiety (Hutcherson et al., 2008; Galante et al., 2014; Seppala et al., 2014; Zeng et al., 2015; Amutio-Kareaga et al., 2017). A previous study framed out a model to contain a wider range of traditional and contemporary meditation practices, categorized into attention, constructive, and deconstructive groups. It worked on the mechanisms of attention regulation and meta-awareness; perspective taking and reappraisal; and self-inquiry. Also, constructive groups aimed to develop healthy interpersonal relationships and positive ethical values that lead to wellbeing. These good and positive relationships with people could expand to loving-kindness and compassion which is associated with wellbeing and emotion (Dahl et al., 2015).

It is assumed that LKM meditators can concentrate on generating loving-kindness and create a sentimental feeling of goodwill with unconditional love. It has been reported that experienced meditators can significantly change their brain activities, especially during compassion meditation (Lutz et al., 2004). The LKM practice follows a Buddhist meditation guidebook, Ven. Buddhaghosa's Visuddhimagga, which provides systematic instructions for LKM (Ñāṇamoli, 1991). A few other studies investigated more modern forms of LKM or compassion meditation (Salzberg, 2002; Hofmann et al., 2011; Galante et al., 2014). Long-term LKM meditators are reported to have a lifelong change of positive psychological characteristics such as empathy, motivation, and honesty. This may partially explain why LKM is known to increase a sense of well-being (Chen et al., 2021).

Neuroimaging studies have found that long-term practitioners of LKM have increased activation in the amygdala, right temporoparietal junction, and right posterior superior temporal sulcus during LKM. In response to emotional stimuli, long-term practitioners of LKM can alter the activation of neural circuitries linked to empathy and the theory of mind (Lutz et al., 2004).

Neuroscientific research can provide more objective evidence on brain reactions to a particular task including meditation (Lutz et al., 2009; Valk et al., 2017). Neurophysiology thus helps to establish a framework model to examine human emotions and mindfulness after meditation (Valk et al., 2017; Don et al., 2022). This model is also comprised of social-affective skills and socio-cognitive skills (Valk et al., 2017). For example, loving-kindness compassion can help individuals tackle difficult and prosocial motivation, while improving their social-affective skills, and this is accompanied by plasticity in frontoinsular regions (Valk et al., 2017). Socio-cognitive skills are related to metacognition of self and others. It might also have influence on inferior frontal and lateral temporal cortices (Valk et al., 2017). This research contributed to the present study by helping to explain the mechanisms at work in meditation.

In the LKM meditation tradition, the practice is mainly related to the heart, which is frequently referred to as the cardiac and body functions, instead of the brain or mind. This is less studied as a majority of the neuroscientific studies are on brain function, although several studies have investigated the potential correlation between meditation practice and cardiac activities. A previous ECG study found that deep Zen meditation can alter heart activity and heart rate variability (Lo and Tian, 2016). An increased correlation between brain and heart is also found during autogenic meditation (Kim et al., 2013, 2014). A more recent neuroimaging study found that meditation is accompanied by specific brain processing and corresponding cardiac rhythms. The interaction between brain networks and cardiac activity may contribute to the fundamental understanding of the neural mechanisms of meditation (Jiang et al., 2020). Concerning compassion meditation, one functional magnetic resonance imaging (fMRI) study revealed that compassion meditation induces a differential correlation between the insula activity and cardiac function between novices and experts LKM meditators (Lutz et al., 2009). This literature, although insufficient, indicates a potential correlation between the brain and heart during LKM meditation.

In this study, by simultaneously measuring brain activity and heart rate, we aimed to better understand the neurophysiological mechanisms and psychological effects of LKM. The purpose of this study was to investigate the effect of LKM on neural activity, body physiology, and their interaction. It is hypothesized that the practice of LKM can induce significant neurophysiological changes, specifically the coherence between heart rate and EEG data.

## Methodology

### Data collection

**Figure 1:**
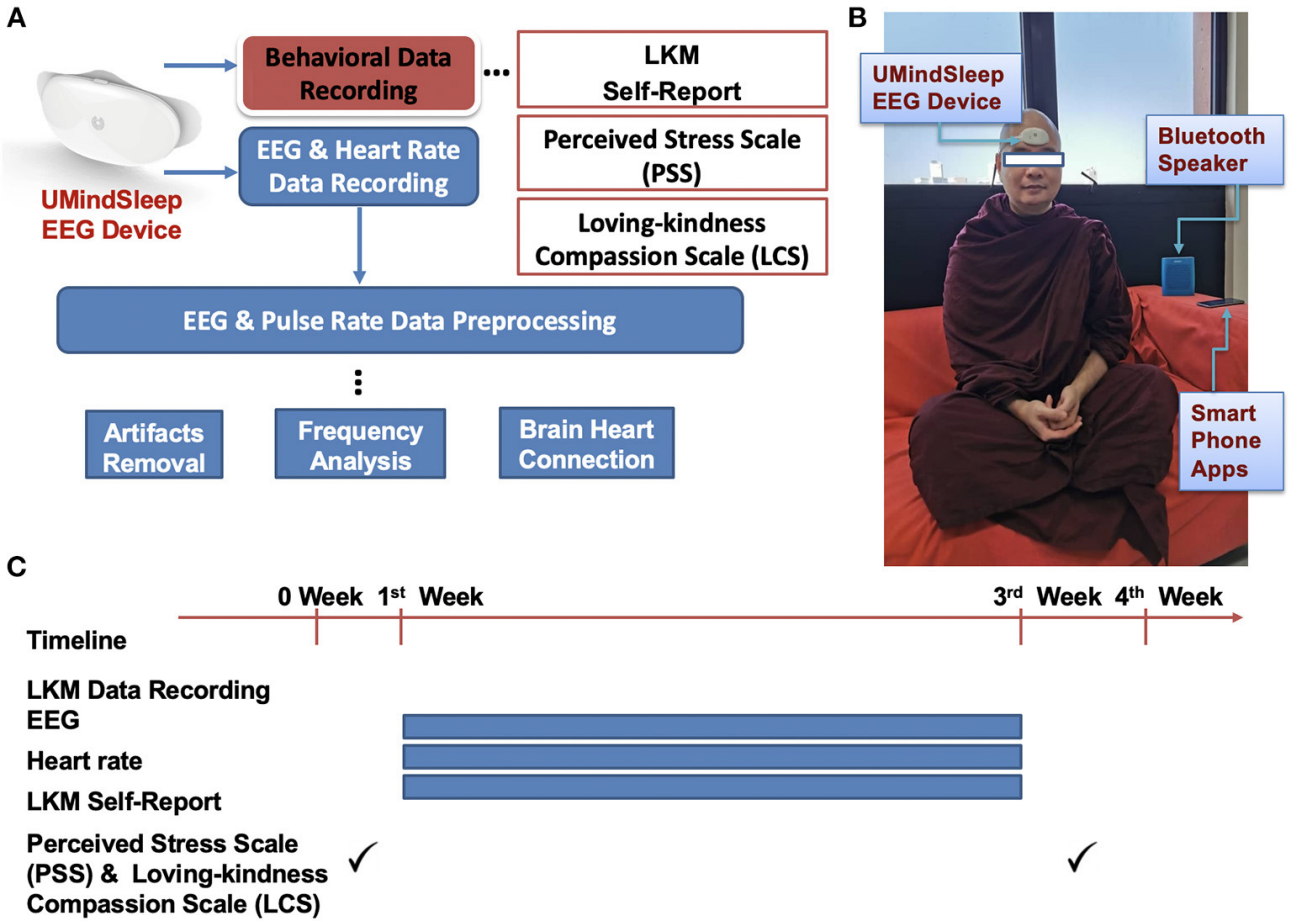
Illustration of data collection and analysis. **(A)** Experimental structure of data collecting. **(B)** A real experimental demonstration. (**C)** The experimental timeline lasted for 3 weeks. Self-report was collected in each session. Perceived Stress Scale (PSS) and Loving-kindness Compassion Scale (LCS) were collected before the first session and after the last session.

A 43-year old long-term meditator from the Theravada Buddhist tradition participated in this study. Using two sets of UMindSleep (EEGsmart) with electrodes placed at Fz and Pz, two EEG Channels were recorded along with heart rate. The sites were selected following preliminary data from a previously unpublished high-density EEG (128 channel) study on LKM, which showed that the frontal and parietal lobes had significant changes in brain activity during LKM practice. The EEG sampling rate was 250 Hz. All EEG signals were referenced to the right mastoid. Data from the device was uploaded to a smartphone *via* Bluetooth. The EEG and pulse rate data were transferred to a computer and then analyzed using EEGLab as a MATLAB toolbox (Delorme and Makeig, 2004) (see Figure 1A).

### Loving-kindness meditation

The participant performed 30 sessions of LKM meditation. Each meditation session started with the participant sitting comfortably with a naturally straight back, with 3~4 deep breaths taken. Then, the forehead was allowed to relax, along with the rest of the body. The sitting posture was Burmese-style posture (easy sitting posture) and sitting on a chair (see Figure 1B).

**Figure 2:**
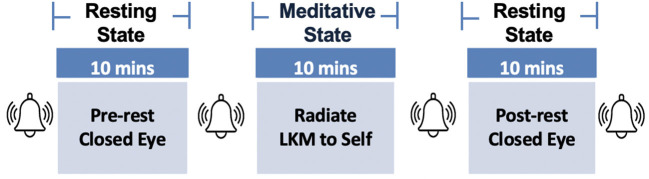
Experimental paradigm constructed of pre-resting, LKM, and post-resting state.

There were three separate conditions the participant performed (see Figure 2): pre-resting task, meditation (LKM), and post-resting task. Each session lasted for 10 min. In the resting tasks, the participant simply rested with his eyes closed while not engaging in any specific mental activity.

During the LKM task, the participant visualized himself with eyes closed and repeatedly radiated loving-kindness toward himself. Simultaneously, the participant silently recited the following set of phrases: “*May I be free from danger and hostility; May I be free from mental suffering; May I be free from physical suffering; May I be happy and well.*,” following the Theravada tradition. LKM practice is in line with the tradition in the book of *Visuddhimagga* (Ñāṇamoli, 1991; Buddhaghosa, 2020).

### Post-LKM self-report

The post-LKM self-report assessment was a specially designed instrument to reflect the outcome of practicing loving-kindness meditation in the Theravada Buddhist tradition (Ñāṇamoli, 1991; Sraman, 2004; Bhaddanta Ācinṇa, 2012; Nyanatusita, 2021). Effectively measuring the progress of LKM practice. Overall progress during meditation was measured with scales which were in line with the awareness concepts of LKM.

With regard to psychological measures, it was important to record the participant's subjective experience with questions composed of psychological measurables. This post-LKM self-report questionnaire consisted of 7 items, including: Body-comfort; Mind-comfort; Body-movement; Radiating-LKM; Visualized-image (envision participant's self-image); Wandering-mind, and Fall-asleep (Thiradhammo, 2014; Anālayo, 2021) (see Supplementary material).

The participant recorded the quality level of each aspect during each task. These measures were used because they described common subjective experiences and obstacles that many LKM meditators experienced. The rating on each item used the Likert scale of 1–9, with 1–3 being “Not Good;” 4–6 being “Neutral;” 7–9 being “Good.” The self-report score was the sum of the 7-item mentioned above in each trial.

### PSS and LCS questionnaires

The effects of LKM were evaluated by comparing the stress level and loving-kindness compassion level. Data were collected at the beginning and the end of the whole experiment (see Figure 1C). The participant's stress level was estimated by the Perceived Stress Scale (PSS), which measured the perception of stress (Cohen et al., 1983). The Loving-kindness Compassion Scale (LCS) was used to evaluate three factors. They were self-compassion, loving-kindness and self-centeredness (Cho et al., 2018).

### EEG processing

EEGLab was used to preprocess EEG and heart rate data during offline analysis. For the EEG analysis, the data was first cleaned of artifacts. Major artifacts including head movement and ocular movement were removed manually. Generally, there were few artifacts as the meditation, pre- and post-LKM resting state were very stable. A notch filter of 50 Hz was used to reduce the contamination of power line interference. The sampling rate was kept at 250 Hz. No segmentation was performed during spectrum EEG analysis, and the whole session of EEG data was analyzed using a Fast Fourier Transform (FFT). The frequency powers were defined as delta (1–4 Hz), theta (4–7 Hz), alpha (8–10 Hz), beta (13–18 Hz), and gamma (25–32 Hz).

### Statistical analysis

The self-assessment recorded the participants' performance quality in each conditional task. Data analysis for this self-report was interpreted by comparing responses in dyads. This included comparing Body-comfort with Body-movement; Mind-relaxation with Wandering-mind; Mind-relaxation with Radiating-LKM; Radiating LKM with Wandering-mind; and Radiating-LKM with Visualized-image. By looking at the data with multiple measures, we were able to get a better idea of the participant's inner experience.

Statistical analysis was performed using the IBM SPSS software (SPSS Inc., Chicago, Illinois, USA). For the EEG frequency power (delta, theta, alpha, beta, and gamma) component, repeated-measure analysis of variance, with time and component as within-subject factors, was used to assess the effects of LKM. Pearson's correlation analysis was used to calculate potential associations between EEG indexes (frequency power), pulse rate, and subjective assessment. The significance level for all statistical analyses was set at *p* < 0.01, adjusted by Bonferroni correction for five EEG spectrum bands.

## Results

**Figure 3:**
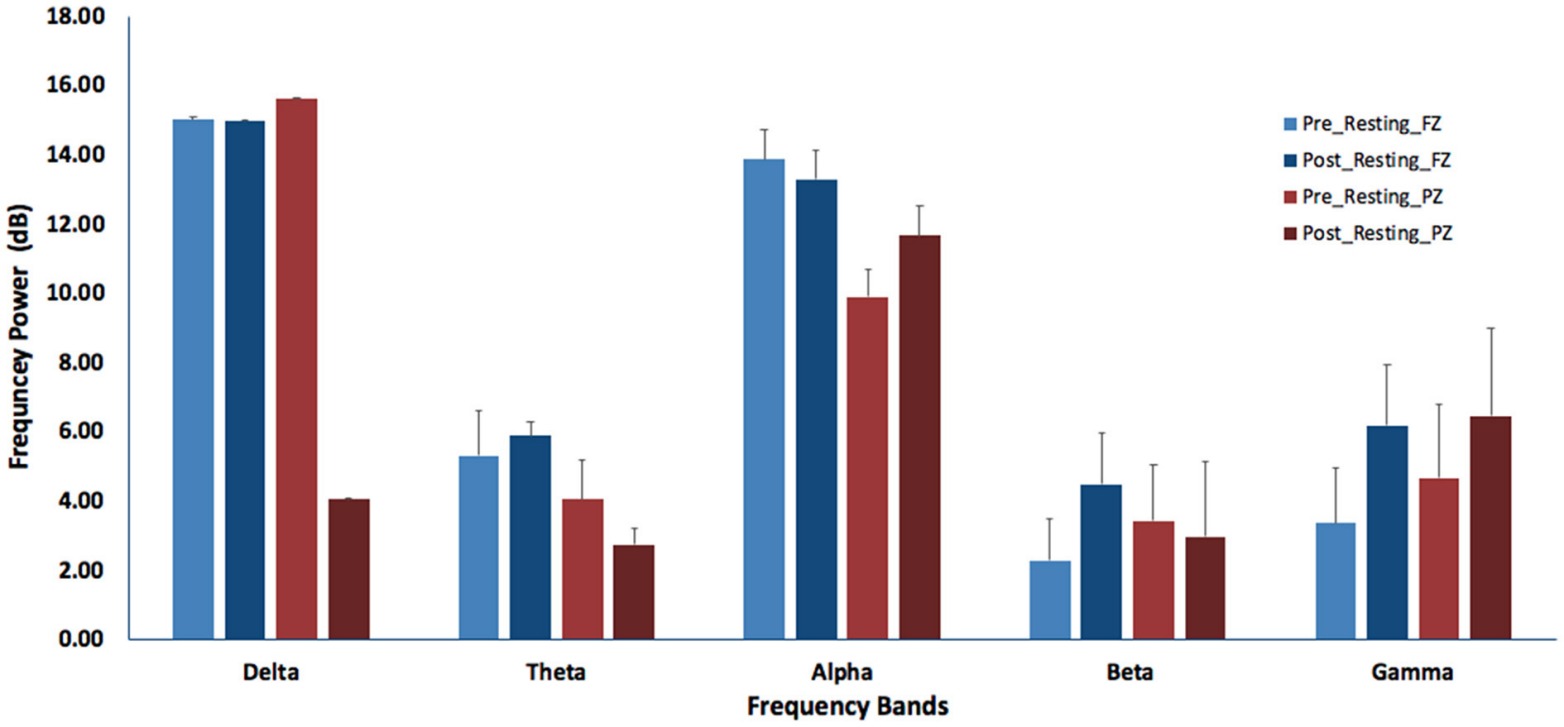
The band-powers of different EEG spectra before and after Loving-Kindness Meditation at Fz and Pz electrode placements.

The results showed that the difference of EEG power between the pre- and post-LKM resting-states were quite significant, with decreased power of Delta band (*t* = 2.387; *p* = 0.024), Alpha band (*t* = 3.261; *p* = 0.003), and increased power of Theta band (*t* =−3.356; *p* = 0.002), Beta band (*t* =−6.311; *p* < 0.001), and Gamma band (*t* =−7.04; *p* < 0.001) at Fz. For the parietal activity, the result at Pz showed decreased power in Delta band (*t* = 3.682; *p* = 0.001), Alpha band (*t* = 4.478; *p* < 0.001), and increased power in Theta band (*t* =−5.199; *p* < 0.001), Beta band (*t* =−6.389; *p* < 0.001), and Gamma band (*t* = −5.8; *p* < 0.001). Heart rate was significantly decreased from pre-resting to post-resting (*t* = 4.092, *p* < 0.001). The self-report score was significantly increased (*t* =−5.215, *t* < 0.001) (see Figure 3).

**Figure 4:**
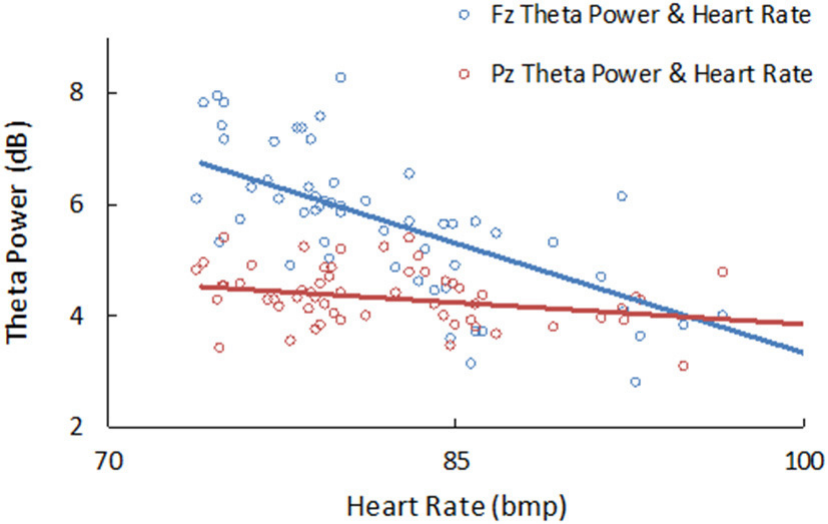
The significant correlation between heart rate and theta power at Fz (*r* = −0.681, *p* < 0.001) and Pz (*r* = −0.349, *p* = 0.008) during LKM task.

Pearson's correlational analysis showed that the heart rate was negatively correlated with theta power at both the frontal placement of Fz and the parietal lobe placement of Pz during pre- and post-resting states, with r = −0.681, *p* < 0.001, and *r* = −0.384, *p* = 0.008, respectively (see Figure 4). At the same time, heart rate was also negatively correlated with alpha power at Fz, *r* = −0.363, *p* = 0.004; and positively correlated with delta power at Fz, *r* = 0.461, *p* < 0.001. Spearman correlation analysis showed a positive correlation between theta power at Fz and the self-designed report score (*r* = 0.580, *p* < 0.01).

There were also significant correlations between Fz theta power and the overall quality of the behavioral assessment of LKM (*r* = 0.601, *p* < 0.001). The overall quality of the behavioral assessment was negatively correlated with heart rate (*r* = −0.401, *p* < 0.001). Interestingly, the alpha band near the frontal lobe at Fz was also correlated with the overall quality of LKM as measured by behavioral assessment (*r* = 0.277, *p* = 0.008).

Perceived Stress Scale (PSS) and Loving-kindness Compassion Scale (LCS) were collected before the first session and after the last session. The results showed that the participant's stress level had slightly decreased (PSS decreased by 3 points), and the Loving-kindness Compassion level increased (LCS increased by 7 points). The PSS score decreased from 25 to 22 indicating stress reduction for the participant. LCS total score increased from 53 to 60 for the participant. All three factors in LCS increased slightly (LCS-Lovingkindness: from 15 to 19; LCS-compassion: from 15 to 20; LCS-Self-centeredness: from 13 to 21).

## Discussion

This pilot study explored the effect of LKM on brain activities and potential brain-heart coherence using wearable devices. In line with the previous study (Jiang et al., 2020), our results revealed that LKM practice has a widespread effect on both the brain and the body.

Among a variety of significant findings in this experienced meditator, theta power was the most sensitive index for LKM training. LKM practice can cultivate a positive attitude which can improve emotion regulation and self-motivation, as revealed by an fMRI study (Kyeong et al., 2017). This fMRI study also demonstrated that LKM can modulate resting-state functional connectivity between the amygdala with the right dorsomedial prefrontal cortex and the left dorsal anterior cingulate cortex. These functional connectivities are correlated with anxiety and depression scales (Kyeong et al., 2017). We suggest that an increased theta power contributes to the cognitive rehearsal of radiating love-kindness to oneself, as theta is synchronized across multiple brain areas during complex cognitive tasks (Ekstrom et al., 2005). These results are in line with other studies on LKM that have found increased theta oscillations during meditation and a positive correlation of theta power with the amount of experience in meditation training (Harne and Hiwale, 2018; Nyhus et al., 2019).

Secondly, we explored the potential body-mind connection, as is much emphasized in traditional Buddhism meditation. We found theta power to be significantly correlated with heart rate. The connection between cerebral and cardiac activities during LKM was also found in a previous fMRI study (Lutz et al., 2009). In that study, a positive coupling of dorsal ACC activity and heart rate was higher during LKM than during the neutral state. This state effect of LKM on brain-heart coupling was stronger for experts than beginners, especially in the right inferior parietal lobe and somatosensory area. This study further suggested that LKM practice can enhance awareness and somatosensory representation of emotion (Lutz et al., 2009).

Thirdly, we found theta power to be significantly correlated with behavioral assessments, which record the quality of mind and body situation during LKM practice. This indicates that the post-LKM self-report could be served as criterion validity of LKM training. A credible subjective report is important in monitoring the progress of LKM practice, because different LKM teachers may have various understandings of LKM techniques, which leads to students having various understandings. Thus, it is difficult to set common standards to assess effectiveness and progress during LKM practice. In the current study, we simplify the behavioral assessment through objective estimations of the quality of both body and mind conditions with two opposing questions: (i.e., comfort and uneasiness). For the body part, an experienced meditator could sit comfortably without much body movement. Moreover, a good LKM meditator can concentrate on LKM by visualizing and radiating love-kindness toward a specific target, without much mind wandering. The mind and body are both comfortable in LKM practice for the experienced meditator. This comfort of mind and body is also referred to as passaddhi in Pali and sometimes translated as calmness or tranquility. It is a key indicator for deeper meditation, according to traditional Buddhism meditation documents (Ñāṇamoli, 1991).

The significant correlation between frontal theta and behavioral scores gives some credit to this simplified behavioral assessment tool as a good estimate of LKM practice. The correlation between frontal brain activity with behavioral scales, is also found in previous studies (Lee et al., 2013; Kyeong et al., 2017), indicating frontal activity plays a key role in relevant behavioral assessments (Hoy et al., 2022; Sriranjan et al., 2022). Interestingly, a recent EEG study also found the religious coping scale to be positively correlated with theta in the right inferior frontal and temporal gyri (Imperatori et al., 2020).

Theta plays an important role in dynamic interactions between different lobes, and this interaction can be modulated by experience and mental training. For example, theta amplitude in frontal-temporal network connectivity is negatively correlated with the duration of meditation experience in a previous study (Jiang et al., 2020). It is further suggested that meditation can alter cortical plasticity in terms of intrinsic reorganization and activity of brain networks. Among the neural plasticity after meditation training, neural representations of visceral activity, especially cardiac activity can be integrated into higher cortical regions associated with cognition and emotion (Jiang et al., 2020).

This study's uniqueness is due to the involvement of subjective assessments on the quality of LKM. This is usually omitted by other research on meditation or mindfulness, as the participants may not know how to assess the quality of meditation. Interestingly, these subjective assessments were strongly correlated with the objective assessments, specifically the theta power. This approach of understanding the subjective data along with the objective data is important for studying LKM, as this meditation in particular, includes the mind and body (mind and heart). This unique finding on EEG, heart rate, and subjective assessments may also help explore neural biomarkers for LKM.

From the Theravada Buddhist perspective, loving-kindness (mettā) tends to proactively follow a positive aspect. It is a positive feeling of kindness and warmth for oneself and sharing with others (Sayadaw et al., 2003; Sayadaw, 2019). On the contrary, compassion (karunā) is more on the passive side. That is, the practitioners tend to perceive people's suffering and want to help them to deal with their adversity (Ñāṇamoli, 1991; Sraman, 2004; Buddhaghosa, 2020). To be more precise, compassion can be defined as perceiving the sufferings of people and being motivated to help (Dahl et al., 2016). Compassion consists of two components which are affective and motivational aspects. In the affective aspect, it involves emotional contagion that induces empathy such as empathic distress and compassion (Singer and Klimecki, 2014; Dahl et al., 2016).

Neff pointed out that self-compassion is related to personal experiences of suffering (Neff, 2003a,b). These sufferings were, for example, encountering failures, inadequacies and pains of life. Self-compassion involves three reactions when painful thoughts and emotions arise. They were a sense of common humanity vs. isolation; mindfulness vs. over-identification; and self-kindness vs. self-judgment. Self-kindness tended to be caring and understanding with oneself (Neff, 2003a,b). When facing unhappiness or dislike, they responded positively with good and supportive words (Neff, 2003a,b). Nevertheless, there is an overlap between LKM and compassion, and compassion arises when one practices loving-kindness meditation (Ñāṇamoli, 1991; Buddhaghosa, 2020). Indeed, the development of these mental states are to reach boundless and further research into the neural mechanisms are open to be explored (Cho et al., 2018; Sirotina and Shchebetenko, 2020; Somaratne, 2022).

This study also demonstrated that a wearable EEG device could be a convenient way to collect neurophysiological data, especially during meditation. The feasibility of EEG devices is vital for meditation data collection, given the nature of meditation. This is in contrast to the time-consuming set-up process and less-comfortable electrical caps of multi-channel EEG, which may affect the comfortability and, subsequently, the quality of meditation training. The advances in neurophysiological technology may finally allow researchers to start exploring more feasible biomarkers associated with ancient meditation techniques due to accumulating data. EEG research with high-density EEG can more accurately illustrate brain activities during meditation, and we have also collected a set of high-density EEG data of LKM. Nonetheless, due to the large variation of meditation methods and different features of neuroimaging technologies, it is important to have a more convenient way to collect bigger datasets, together with subjective assessments of both body and mind, in order to better monitor the neural dynamics of meditation. Our results may imply that the theta frequency and its correlation with cardiac activity could potentially be a neural biomarker during LKM practice.

There are several limitations worth noting in the current study. First, since there was only one experienced LKM meditator, the results lack duplicate detection and therefore further implications of the study are limited. Another limitation is that the wearable EEG device is susceptible to artifacts of body movement, ocular activity and potentially other environmental influences. These artifacts are not easily removed with single-channel EEG data, as traditional algorithms such as independent component analysis (ICA) cannot be applied to single-channel EEG data. Independent component analysis (ICA) together with singular spectrum analysis (SSA) may help with artifact suppression in one-channel EEG data.

## Conclusion

In summary, this study found that LKM can significantly modulate brain activities before and after meditation for a long-term practitioner. More importantly, EEG changes after LKM, in theta power were found to be significantly correlated with the physiological change of heart rate, along with the subjective assessments. These results indicate that the theta band and its correlation with heart rate are sensitive to the effect of LKM meditation. In the future, groups of long-term meditators, as well as laymen with high-density EEG could be recruited to validate the current findings.

## Data availability statement

The raw data supporting the conclusions of this article will be made available by the authors, without undue reservation.

## Ethics statement

The studies involving human participants were reviewed and approved by the Human Research Ethics Committee (HREC) of the University of Hong Kong (No. EA210145). The patients/participants provided their written informed consent to participate in this study. Written informed consent was obtained from the individual(s), and minor(s)' legal guardian/next of kin, for the publication of any potentially identifiable images or data included in this article.

## Author contributions

GW was involved in collecting data and methodology design and writing. RS provided software program and data analysis. JA helped writing and proofreading. KY helped proofreading. SY helped on device technique support and data analysis. JG methodology helped on design and writing. All authors contributed to the article and approved the submitted version.

## Funding

This study was partially supported by Hong Kong Council of Early Childhood Education and Services, Anthony Sweeting Memorial Fund (Donated by Ms. SanSan Ching) and the Malaysian Chamber (HK & Macau) Students Trust Fund (MAYCHAM).

## Conflict of interest

Author SY was employed by Shenzhen EEGSmart Technology Co., Ltd. KY studied at The Buddha Dharma Centre of HK. The remaining authors declare that the research was conducted in the absence of any commercial or financial relationships that could be construed as a potential conflict of interest.

## Publisher's note

All claims expressed in this article are solely those of the authors and do not necessarily represent those of their affiliated organizations, or those of the publisher, the editors and the reviewers. Any product that may be evaluated in this article, or claim that may be made by its manufacturer, is not guaranteed or endorsed by the publisher.
