# Supplementary material for: Loving-kindness meditation (LKM) modulates brain-heart connection: An EEG case study
Source: Front Hum Neurosci. 2022 Sep 1;16:891377. doi: 10.3389/fnhum.2022.891377 (PMC9477156; doi:10.3389/fnhum.2022.891377)
Supplement: Supplementary file 1 [file Data_Sheet_1.docx]

Supplementary material: **Description of Post- LKM Self-report**

**Meditation Date: (dd/mm/yy) Participant’s Record File Name:**

**Experiment Time : From （a.m./p.m.） to: （a.m./p.m.）**

**Note: Ranking from 1 – 9 (Not Good [1 – 3]; Neutral [4 – 6]; Good [7 – 9]) or N/A**

**Do you think during the LKM experiment everything go well? 1- 9 ( )**

| **Paradigm**  **Condition Tasks** | **Pre-Rest** | | | | | **Radiating LKM** | | | | | **Post-Rest** | | | | |
| --- | --- | --- | --- | --- | --- | --- | --- | --- | --- | --- | --- | --- | --- | --- | --- |
| **Record No:** |  |  |  |  |  |  |  |  |  |  |  |  |  |  |  |
| **Body-comfort** |  |  |  |  |  |  |  |  |  |  |  |  |  |  |  |
| **Mind-comfort** |  |  |  |  |  |  |  |  |  |  |  |  |  |  |  |
| **Body-movement** |  |  |  |  |  |  |  |  |  |  |  |  |  |  |  |
| **Radiating-LKM** | **N/A** | | | | |  |  |  |  |  | **N/A** | | | | |
| **Visualized-image** |  |  |  |  |  |  |  |  |  |  |  |  |  |  |  |
| **Wandering-mind** |  |  |  |  |  |  |  |  |  |  |  |  |  |  |  |
| **Fall-asleep** |  |  |  |  |  |  |  |  |  |  |  |  |  |  |  |

Body-comfort was the first item which referred to the quality of tranquility of body (Anālayo, 2021; Bhaddanta Āciṇṇa, 2012). Also, it is related to the level of Body-movement. When the quality of body tranquility increased, constant change of sitting posture due to physical discomfort would be reduced, therefore reducing body-movement. In some cases, the quality of mental focus during the meditation practice could be affected when the practitioner’s body was not comfortable during LKM meditation.

Similarly, when physical calmness increased, physical movement decreased. At this point, peace of mind aroused— mind comfort. Mind comfort (tranquility of mind) was also related to Wandering-mind. The Wandering-mind has been considered one of the five hindrances in meditation practice (Bhaddanta Āciṇṇa, 2012; Thiradhammo, 2014). The wandering-mind, aroused by restlessness and remorse or doubt. This might cause the quality of mind-comfort level to decrease. Eventually, affecting the quality of focus on meditation practice. When we came to the items of Radiating-LKM and Visualized-image, this was another pair of comparative items. The practitioner needed to have a target for radiating LKM (Ñāṇamoli, 1991; P. A. T. Sayadaw, 2019; Sayadaw, Revata, & Dhammasubho, 2003). Visualizing an object with a clear smiling image with eyes closed, followed by Radiating-LKM onward.

Radiating-LKM with clear consciousness could facilitate to sustain the quality of awareness for mindfulness. As well, the frequency of conscious perception on loving-kindness might increase. Eventually, the practitioner would have more experiences of calmness and stability. The practitioner might rank in Good when one could stably radiate LKM continuously without breaking. The item of Fall asleep, is one of the main five hindrances of meditation. This was called as “sloth and torpor”. The practitioner lost the awareness and consciousness. During the experiment, the practitioner needed to rate the time duration or number of time if they fall asleep during the experimental task. For example, if the mind was aware, the practitioner might rank in Good (7 – 9). If it happened more than 5-times, ranking could be (4 – 6). If it often happened, and more than half of the time during the experiment, ranking range was considered as (1 – 3). The Fall asleep item was in comparison with all the other items.

During each task, the participants recorded the level of quality for every item which measured and described common subjective experience and obstacle that many LKM meditators experience on the self-report. The rate on each item used the Likert scale with range of 1-9. Scale of 1-3 indicated ‘Not Good’; 4-6 indicated ‘Neutral’ and 7-9 indicated ‘Good’. In each scale category, there were 3-Likert points. For example, Not Good [1 – 3], if the grade (1) indicated “Not Good” scale was severe; grade (2) indicated the scale was moderate and grade (3) indicated the “Not Good” scale was not severe. Similarly, it applied the same logic to the other two scale categories of being “Neutral” and “Good”. This implied the higher the score which the participants rate, the good feeling the participants had experienced and vice versa. For instance, scale (9) represented good in great. At the end, the sum of the 7-item scores generated the post-experimental self-report score.
